# The potential of real-time fMRI neurofeedback for stroke rehabilitation: A systematic review

**DOI:** 10.1016/j.cortex.2017.09.006

**Published:** 2018-10

**Authors:** Tianlu Wang, Dante Mantini, Celine R. Gillebert

**Affiliations:** a Department of Brain & Cognition, University of Leuven, Leuven, Belgium; b Department of Experimental Psychology, University of Oxford, Oxford, United Kingdom; c Research Center for Movement Control and Neuroplasticity, University of Leuven, Leuven, Belgium; d Department of Health Sciences and Technology, ETH Zurich, Zurich, Switzerland

**Keywords:** fMRI, Neurofeedback, Brain injury, Neuropsychology, Rehabilitation, Stroke

## Abstract

Real-time functional magnetic resonance imaging (rt-fMRI) neurofeedback aids the modulation of neural functions by training self-regulation of brain activity through operant conditioning. This technique has been applied to treat several neurodevelopmental and neuropsychiatric disorders, but its effectiveness for stroke rehabilitation has not been examined yet. Here, we systematically review the effectiveness of rt-fMRI neurofeedback training in modulating motor and cognitive processes that are often impaired after stroke. Based on predefined search criteria, we selected and examined 33 rt-fMRI neurofeedback studies, including 651 healthy individuals and 15 stroke patients in total. The results of our systematic review suggest that rt-fMRI neurofeedback training can lead to a learned modulation of brain signals, with associated changes at both the neural and the behavioural level. However, more research is needed to establish how its use can be optimized in the context of stroke rehabilitation.

## 1 Introduction

The number of stroke survivors is continuously increasing with the ageing of the population: about 15 million people worldwide suffer from stroke every year, of whom 5 million die, whereas another 5 million become chronically disabled (WHO, 2012). Behavioural deficits in cognitive and motor domains are highly prevalent and persistent in stroke survivors (Bickerton et al., 2014, Demeyere et al., 2015, Demeyere et al., 2016, Jaillard et al., 2009, Planton et al., 2012, Verstraeten et al., 2016). Neurophysiological and neuroimaging studies suggested that stroke causes network-wide changes across structurally intact regions, directly or indirectly connected to the site of infarction (Carrera and Tononi, 2014, Carter et al., 2010, Gillebert and Mantini, 2013, Grefkes et al., 2008, Ward and Cohen, 2004). Disruptions in even one of the many networks or brain regions implicated in the different aspects of motor function and cognition can have a major impact on quality of life (Achten et al., 2012, Hochstenbach et al., 1998). Accordingly, both local tissue damage and secondary changes in brain function should be considered when developing rehabilitation strategies to improve the recovery rate and generally increase the quality of life in stroke survivors (Chechlacz et al., 2015, Chechlacz et al., 2013, Corbetta et al., 2015, Gillebert and Mantini, 2013). In this regard, the use of neurofeedback may be a promising approach.

### 1.1 Neurofeedback

Neurofeedback works as a closed loop system that provides real-time information regarding the participant's own brain activity and/or connectivity, which can be used to develop self-learning strategies to modulate these brain signals (Weiskopf, Mathiak, et al., 2004). It follows the principle of operant conditioning, a method of learning that occurs through reinforcing specific behaviour with rewards and punishments (Skinner, 1938). If the participant learns to control activity of the brain areas targeted through neurofeedback, this may ultimately lead to a measurable behavioural change that is related to the function of those areas (DeCharms et al., 2005, Haller et al., 2010, Hartwell et al., 2016).

The origins of neurofeedback are rooted in electroencephalography (EEG), which measures dynamic changes of electrical potentials over the participant's scalp (Nowlis & Kamiya, 1970). This technique is portable and inexpensive, and provides estimates of brain activity at high temporal resolution. EEG neurofeedback has been widely used over the years to induce long-lasting behavioural changes, both in healthy volunteers and in patients (Gruzelier, 2014, Nelson, 2007). However, because of the low spatial resolution associated with this technique, it is very challenging to selectively target brain areas of interest. As such, the effects of EEG neurofeedback are often not specific (Rogala et al., 2016, Scharnowski and Weiskopf,). Other neuroimaging techniques used for neurofeedback include magnetoencephalography (MEG) (Buch et al., 2012, Okazaki et al., 2015) and functional near-infrared spectroscopy (fNIRS) (Kober et al., 2014, Mihara et al., 2013). However, as also for EEG, their spatial resolution is relatively limited and they do not permit to target precise brain regions.

**Fig. 1:**
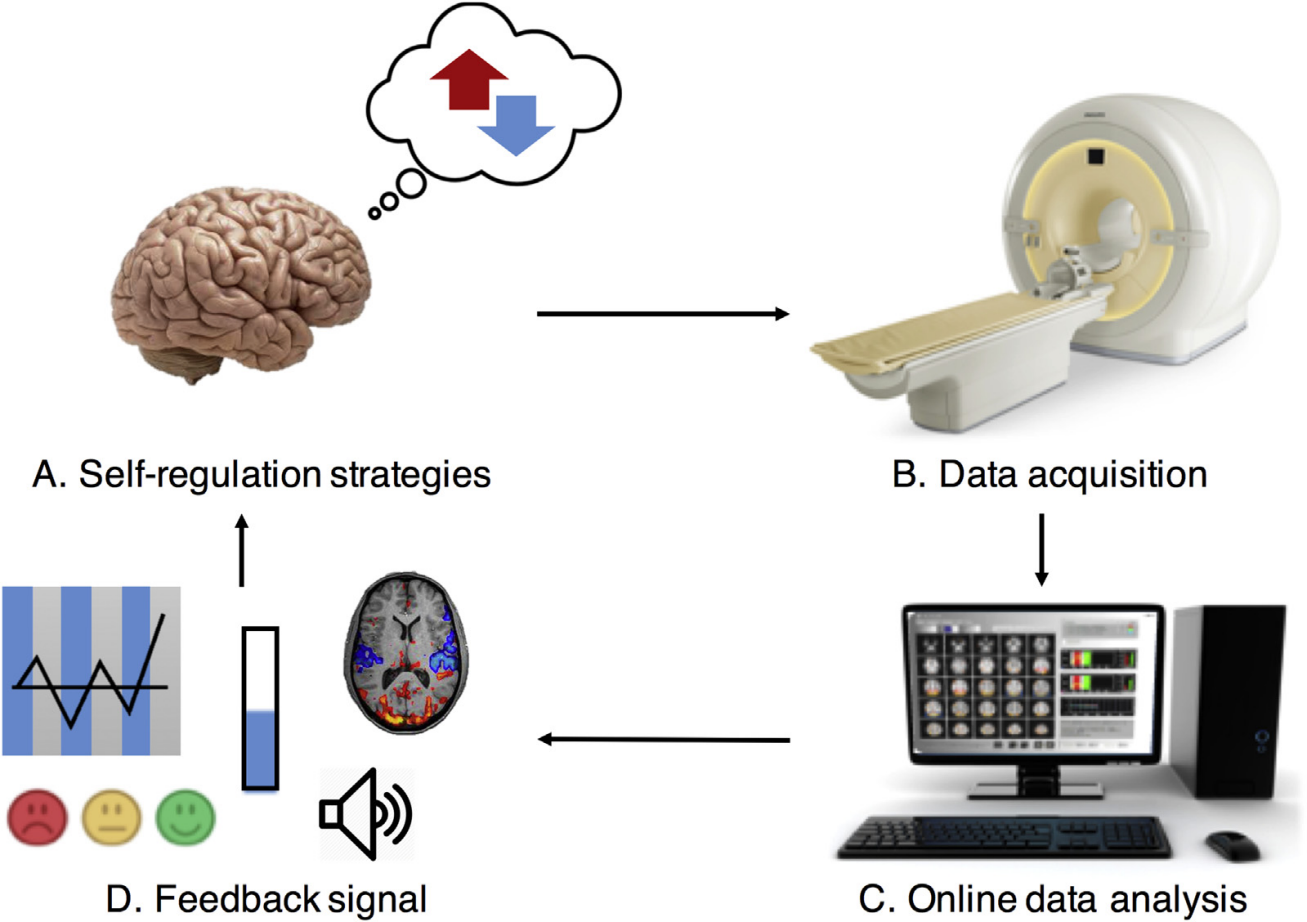
Real-time fMRI neurofeedback is a closed-loop system that can be used to voluntarily modulate brain-activity through the principle of operant conditioning. (A) The participants use self-generated or prior instructed strategies to attempt to change their brain activity. (B) fMRI data are acquired and (C) processed in real-time. Computer programs select the relevant signals and (D) return these to the participants after varied degrees of pre-processing to allow them to adjust their control strategies.

The field of neurofeedback has rapidly developed and delved into new avenues by the introduction of real-time functional magnetic resonance imaging (rt-fMRI) technology (Cox, Jesmanowicz, & Hyde, 1995). Accordingly, in the past years there has been a steady increase of studies focussing on rt-fMRI neurofeedback applications to induce behavioural changes (Sulzer et al., 2013). Rt-fMRI neurofeedback uses the blood-oxygenation level-dependent (BOLD) signal to present contingent feedback to the participant and to enable modulation of brain activity (Fig. 1). Various acquisition parameters are available, and chosen based on a trade-off between spatial and temporal resolution, and signal-to-noise ratio (Weiskopf, Scharnowski, et al., 2004). The analysis is performed almost immediately or with a delay of a few seconds depending on the available computational resources. With a much higher spatial resolution than EEG, fMRI allows for a refined delineation of both cortical and subcortical target regions. These properties can be valuable for neurofeedback applications (Stoeckel et al., 2014). Recent studies suggest that rt-fMRI is a mature technology to use in the context of neurofeedback training (for a review, see e.g., Ruiz et al., 2014, Weiskopf, 2012). As a result, doors are being opened to the application of rt-fMRI neurofeedback in ameliorating disrupted brain functions in stroke survivors.

### 1.2 Stroke rehabilitation

The last two decades have witnessed a proliferation of rehabilitation strategies to promote functional recovery after stroke, such as task-specific exercises, task repetition, mental and motor imagery, imitation and – among technological approaches – robot-assisted training, muscle stimulation, magnetic and electrical stimulation, and the use of virtual environments (Cicerone et al., 2000, Loetscher and Lincoln, 2013). However, none of these approaches has yet yielded satisfactory results. Most likely, this is because they do not properly account for the structural, metabolic, and electrophysiological consequences of stroke, and are based on theories of neural plasticity that mainly focus on damage and reorganization of local circuitry, without considering brain-wide effects (Baldassarre et al., 2014, Langhorne et al., 2011). Furthermore, current rehabilitation protocols do not sufficiently account for across-subject variability. Large across-subject differences have indeed been reported in the type and degree of behavioural impairment and in the spontaneous functional reorganization after stroke (Gillebert and Mantini, 2013, Stoeckel et al., 2014). Based on these considerations, it could be argued that rt-fMRI neurofeedback may be effective for reducing stroke-induced behavioural deficits because the feedback is based on individual brain dynamics, and the brain signals can be derived at high spatial resolution.

### 1.3 Objectives of the systematic review

This systematic review examines whether rt-fMRI neurofeedback can induce *neural* and *behavioural* changes related to motor function or cognition. It thereby evaluates the potential of rt-fMRI neurofeedback-based therapy for stroke rehabilitation. More specifically, we aim to (1) provide an overview of empirical studies investigating the effectiveness of rt-fMRI neurofeedback in modulating brain function and behaviour in healthy individuals and stroke survivors; (2) evaluate the quality of the studies against pre-set methodological and theoretical criteria; (3) provide indications for investigating the use of rt-fMRI neurofeedback in the field of stroke rehabilitation.

## 2 Methodology

### 2.1 Search methods

We searched 4 databases (Web of Knowledge/Web of Science, Pubmed, Scopus, and the recently released Real-time Functional Imaging and Neurofeedback (rtFIN) literature database (http://www.rtfin.org/literature.html)) from 1970 to July 2017, and screened reference lists. We used the following keywords: FMRI AND (real-time OR neurofeedback) AND (stroke OR cognition OR attention OR memory OR perception OR language OR motor OR behaviour). For the rtFIN database, we searched for relevant studies by selecting the categories *fMRI* and *Multiple modalities*.

### 2.2 Inclusion and exclusion criteria

We sought all studies in which the aim was to use real-time fMRI neurofeedback to modulate brain activity, connectivity, and/or the ensuing behaviour related to cognition and motor function in healthy individuals and/or stroke survivors. We restricted the search to the motor and cognitive domains, as these have been shown to be frequently affected after stroke (Hochstenbach et al., 1998, Langhorne et al., 2009). Studies evaluating patients with progressive brain diseases, neurodevelopmental or neuropsychiatric disorders were not included. Due to the novelty of this field, we also retained studies with small sample sizes that were labelled as feasibility, proof-of-concept, or pilot studies. We only considered published manuscripts in English.

### 2.3 Outcome measures

Two outcome variables were considered in the study. The first involved measures of learned self-regulation of brain function (Sulzer et al., 2013), as assessed by the activation level in the target region-of-interest (ROI) or across the brain, or the functional connectivity between two or more ROIs (Sulzer et al., 2013). The second outcome variable involved measures of behavioural change in cognitive and motor domains. For any of the aforementioned outcome measures, successful learning can be inferred from comparing participants who received neurofeedback to participants who did not receive real feedback (sham-neurofeedback). Alternatively, it can be inferred from within-group comparisons between neurofeedback training runs and transfer runs (i.e., runs during which no feedback is presented) (Weiskopf, Scharnowski, et al., 2004).

### 2.4 Quality assessment

Two experimenters (TW and CRG) independently assessed the methodological quality of the studies according to the Joanna Briggs Institute (JBI) critical appraisal tools (JBI, 2016). We used the checklist for quasi-experimental studies, which includes 9 criteria (established temporal relationship between the variables; similar participants; similar treatment; control group; multiple outcome measurements; follow-up; similar outcome measurements; reliable outcome measurements; and appropriate statistical analysis). One point was given for the fulfilment of each of the criteria above. Studies scoring between 0 and 3, and those scoring between 4 and 6 were considered to be of low and moderate quality, respectively. If a study scored a 7 or higher, it was considered a high quality study (Luctkar-Flude & Groll, 2015).

## 3 Results

**Fig. 2:**
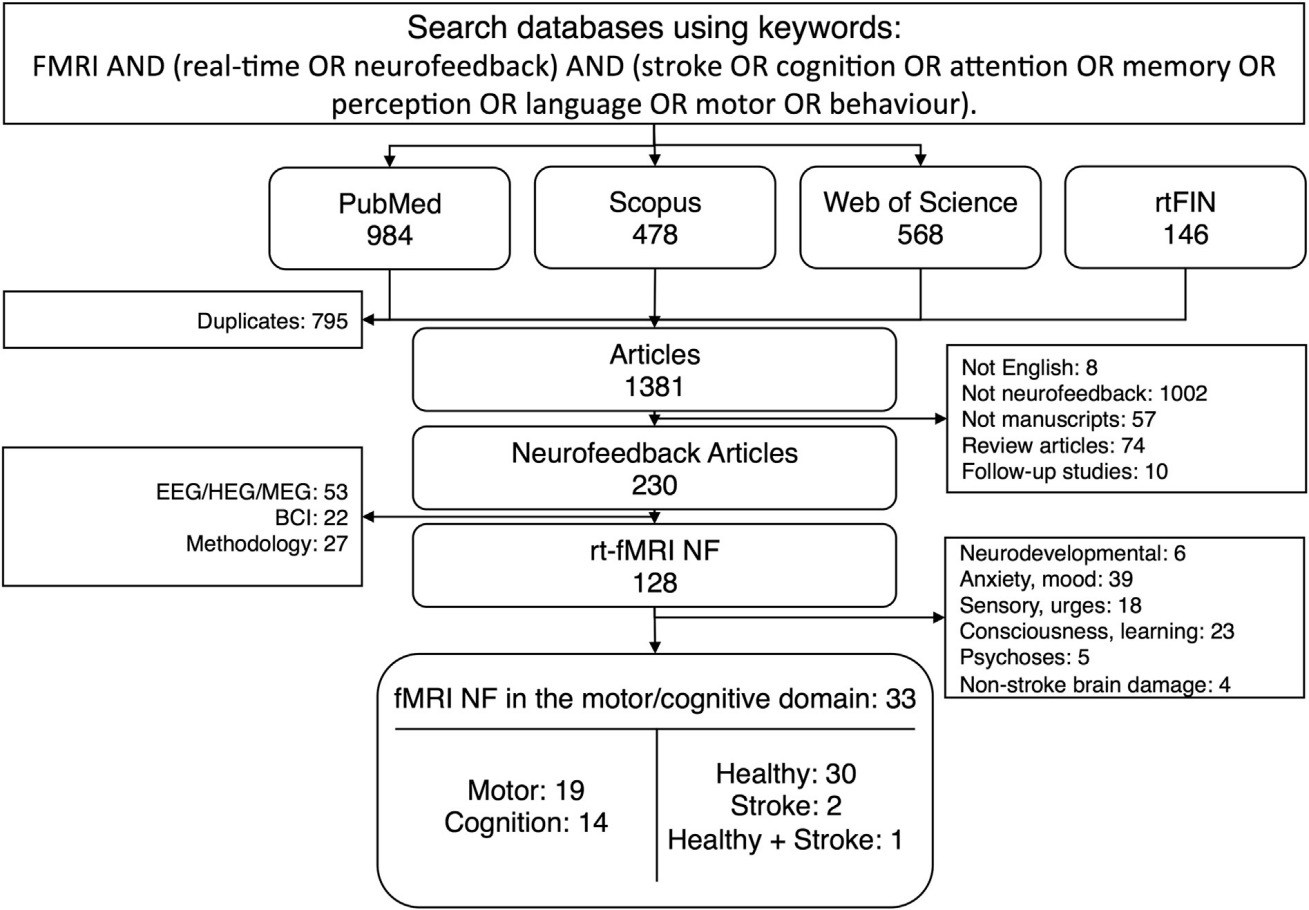
The search decision flow diagram shows the selection process of the 33 papers included in this systematic review. *Abbreviations*: rtFIN, real-time Functional Imaging and Neurofeedback; NF, neurofeedback; EEG, electroencephalography; HEG, hemoencephalography; MEG, magnetoencephalography; BCI, brain computer interface.

Application of inclusion and exclusion criteria led to the identification of 33 studies that used rt-fMRI neurofeedback in healthy participants and/or stroke survivors (Fig. 2). These studies included a total of 651 healthy participants and 15 stroke survivors. The total sample size per study ranged between 4 and 80 individuals. The age of the healthy participants ranged between 18 and 77, and the age of the stroke patients between 41 and 75 years. The targeted domain, the presence of a control group, the duration and planning of the neurofeedback training, and the assessment of training outcomes differed considerably between the studies and are summarized in Table 1, Table 2. About half of the studies (*N* = 17) explicitly examined the effect of rt-fMRI neurofeedback on behavioural outcome measures (Table 2).

**Table 1:** Overview of the studies examining the effect of rt-fMRI neurofeedback on neural measures only.^a^

Study	ROI(s) and definition	Participants^b^^,^^c^	Training sessions and feedback	Results	Quality
**Motor domain**					
Auer et al. (2015)	Bilateral M1; functional localizer	32 young healthy adults (16 controls)	• 24 runs of 5.8 min each, 12 days over 4 weeks; • Continuous horizontal bar • No NF training for the controls	• Significant transfer of self-regulated control in most of the participants, with a high spatial specificity to the ROI	High
Berman et al. (2012)	Left M1; functional localizer	15 young healthy adults (no controls)	• 2–4 runs of 4 min each, 1 day • Continuous thermometer with target line	• Successful up-regulation of ROI activity during both NF and transfer runs with motor execution, but not with motor imagery	Moderate
DeCharms et al. (2004)	Left M1 and S1; functional localizer	9 young healthy adults (3 controls)	• 3 runs of 20.5 min, 1 day • Continuous line graph, or virtual reality interface of a corresponding dynamic virtual object • Sham NF from a background region at an earlier time-point in the same session	• Successful regulation of ROI activity, specific to the experimental group	High
Hampson et al. (2011)	Bilateral SMA; functional and anatomical localizer	8 young healthy adults (no controls)	• 24 runs over 2 weeks • Continuous line graph	• Successful regulation of ROI activity in sessions 2–4, but no significant increase over the sessions • Decreased connectivity between the SMA and subcortical regions following training	Moderate
Johnson et al. (2012)	Left PMC; functional localizer	13 young healthy adults (no controls)	• 4 runs of 10.3 min each, 1 day; • Continuous or intermittent thermometer	• Participants preferred intermediate over continuous feedback • PSC differences more significant in the intermittent than the continuous condition	Low
Liew et al. (2016)	Left M1 and thalamus; functional localizer	4 elderly chronic stroke patients with right hemiparesis (no controls)	• 18 ± 3 runs of 4 min each, 2 days • Continuous thermometer	• Increased connectivity between the start and the end of the NF training in 3/4 participants • All participants showed an increased cortical–subcortical resting state connectivity • Individuals with greater motor impairment showed larger increases in learned self-modulation	Moderate
Marins et al. (2015)	Left PMC; anatomical localizer	28 young healthy adults (14 controls)	• 3 runs of 6.5 min each, 1 day • Continuous vertical bar • Controls receive random signals ‘without meaning, displayed for experimental purposes’	• Increased activation in the ROI in the last NF run compared to the first run • Associated increases in activity of motor control regions, not present in the control group	High
Neyedli et al. (2017)	Bilateral M1; functional localizer	26 young healthy adults, (13 controls); 18 elderly healthy adults (9 controls)	• 4 runs of 6 min each, 1 day • Continuous horizontal bar • Sham NF from a non-activated region	• Young and older adults increased their lateralized activity between the motor cortices • Only young adults could maintain the lateralized activity during transfer	Moderate
Perronnet et al. (2017)	Left M1; functional localizer	10 young healthy adults (no controls)	• 3 runs of 6.7 min each, 1 day • Moving ball	• Unimodal fMRI-NF and bimodal EEG–fMRI-NF in a motor regulation task aided in learning self-regulation • Motor imagery-related haemodynamic and electrophysiological activity are both modulated during EEG-, fMRI- as well as EEG-fMRI-NF	Moderate
Xie et al. (2015)	Right dorsal PMC; functional localizer	24 young healthy adults (12 controls)	• 4 runs of 7.5 min each, 1 day • Continuous line graph • Sham NF from the experimental group	• Associated decrease in connectivity between bilateral PMC and right posterior parietal lobe	High
Yoo and Jolesz, 2002	Left M1 and S1, parts of pre-motor areas; functional and anatomical localizer	5 young healthy adults (no controls)	• 1 run of 8 min, 1 day • Intermittent statistical map of pixel-by-pixel brain activity	• All achieved a 3-fold increase in the number of activated voxels in motor and somatosensory areas	Low
Yoo et al. (2008)	Left M1; functional and anatomical localizer	24 young healthy adults (12 controls)	• 7 runs of 1.2 min, 1 day; follow-up after 2 weeks • Continuous line graph • Sham NF from a non-activated region in an earlier session	• Successful regulation of ROI activity, retained after a 2 week long daily practice without NF • Recruitment of additional circuitries implicated in motor skill learning, unique to the experimental group	High
**Cognitive domain**					
Banca et al. (2015)	hMT+/V5 complex; functional localizer	20 young healthy adults (no controls)	• Self-paced training session, 1 day • Auditory feedback between 0 (lowest) and 5 (highest)	• Successful regulation of ROI activity through focused visual motor imagery in most of the participants • Recruitment of a novel circuit including putative V6 and medial cerebellum	Moderate
Ramot et al. (2016)	FFA and PPA; functional localizer	16 young healthy adults (no controls)	• 25 runs of 10 min each over 5–7 days • Auditory feedback with positive/negative sounds	• Induced modulation of FFA/PPA or PPA/FFA activity ratio in 10/16 participants without them being aware • Associated changes in functional connectivity in the auditory cortex	Moderate
Yoo et al. (2006)	Left primary and secondary auditory areas; anatomical and functional localizers	22 young healthy adults (11 matched controls)	• 5 runs, 40 min total, 1 day • Intermittent auditory feedback of PSC • No neurofeedback information for the controls	• Required target level of regulation (40% increase from baseline) reached by 10/11 resp. 7/11 experimental and control participants • No significant difference between the pre- and post-training scans in either group • The experimental group showed a significant increase in activated volume and BOLD signal in the last NF run	High
Yoo et al. (2007)	Primary and secondary auditory areas; functional localizer and neuroanatomical template	24 young healthy adults (12 matched controls)	• 7 runs of 1.8 min each, 1 day; Follow-up after 2 weeks of self-practice • Continuous line graph • Sham NF from non-activated regions, scrambled in the time-domain	• Enhanced activity during NF in attention-related regions, reduced activity in regions part of resting-state networks, maintained after 2 weeks of self-training • Modulation of connectivity during NF, no significant changes between the pre- and post-training, but more significant after 2 weeks of self-training	High

a Abbreviations in alphabetical order: BOLD, blood-oxygen level dependent; FFA, fusiform face area; hMT+/V5, middle temporal visual cortex; M1, primary motor cortex; NF, neurofeedback; PMC, premotor cortex; PPA, parahippocampal place area; PSC, percent signal change; ROI, region of interest; S1, primary somatosensory cortex; SMA, supplementary motor area.

b Where applicable, the number of controls is included in the total number.

c The age of young adults ranged from 18 to 46; the age of elderly adults ranged from 41 to 77.

**Table 2:** Overview of the studies examining the effect of rt-fMRI neurofeedback on neural and behavioural outcome measures.^a^

Study	ROI(s) and definition	Participants^b^^,^^c^	Training sessions and feedback	Results	Quality
**Motor domain**					
Blefari et al. (2015)	Left M1; anatomical and functional localizers	14 young healthy adults (no controls)	• 3 runs of 6 min each, 1 day • Continuously vertically moving ball	• Left M1 activity was lower during neurofeedback • Isometric pinching task showed no change during pre- and post-training • Correlations between left M1 activation and performance	Moderate
Bray et al. (2007)	Left M1 and S1; functional localizer	40 young healthy adults (9 controls)	• 4 runs of 8 min each, 1 day • Intermittent feedback, monetary reward • Sham NF from the experimental group	• Overall brain activity increase in the NF group, and no significant change in the control group • Participants receiving NF showed significant faster reaction times with a coherent cue	High
Chiew et al. (2012)	Bilateral M1; functional localizer	18 young healthy adults (5 controls)	• 4 runs of 8.5 min each, 1 day • Continuous arrow vector; length represents brain activity • Sham NF from the experimental group	• Increased laterality index between left and right M1 in 6/13 NF participants • Button press reaction time test showed no difference pre- and post-training in both NF and sham-feedback groups.	High
Hui et al. (2014)	Right PMC; functional localizer	28 young healthy adults (13 controls)	• 4 runs of 7.5 min each, 1 day • Continuous line graph • Sham NF from the experimental group	• Significant correlation between changes in ROI activity in the last run and network connectivity • Significant increased performance in finger tapping task in both groups, but only correlated with functional connectivity in the NF group	High
Scharnowski et al. (2015)	SMA and PHC; functional localizer	7 young healthy adults (no controls)	• 12–22 runs of 8 min each, 4–6 days; • Continuous graph of differential SMA–PHC or PHC–SMA signal	• Significant increases in differential feedback signal associated with training, maintained in the absence of neurofeedback in transfer runs • Increased negative coupling between SMA and PHC • Improved reaction times during the motor task correlated with SMA activity, and performance in word memory correlated with in PHC activity	Low
Sitaram et al. (2012)	PMv; functional localizer	2 elderly chronic stroke patients with right hemiparesis 4 young healthy controls	• 10 runs of 7.5 min each over 3 days • Continuous video feedback during runs 1–2, continuous thermometer feedback in the remaining runs	• Increased ROI activity and decreased intracortical inhibition over the course of the training • The visuomotor pinch-force task showed improved performance across trials in 1 patient and 3 healthy participants	Moderate
Zhao et al. (2013)	Right dorsal PMC; functional localizer	24 young healthy adults (12 controls)	• 4 runs of 7.5 min each, 1 day • Continuous line graph • Sham NF from the experimental group	• Increase in connectivity from the dorsal PMC to other motor-related areas in the experimental group and progressive decrease in the control group • Significant improvements in the behavioural finger tapping task, higher in the experimental compared to the control group	High
**Cognitive domain**					
Amano et al. (2016)	V1/V2; functional localizer for fMRI decoder	18 young healthy adults (6 controls)	• 3 runs on 3 days; • Intermittent visual disc size • No NF training for the controls	• Induced associative learning between colour and grating orientation in the early visual cortex (V1/V2) • Assessed with a forced-choice test after training, persisting for 3–5 months after training	High
DeBettencourt et al. (2015)	Frontoparietal attention network, functional localizer	80 young healthy adults (Experimental group + 4 control groups, 16 subjects each)	• 3 runs of max 2 h each, 3–5 days • Composite faces/scenes stimuli, proportion of task-relevant information related to how well the participant paid attention • Sham NF from the experimental group • No-NF: no feedback, outside the scanner • RT-feedback: response time feedback, outside the scanner • RT-sham control: random feedback from the RT-feedback group	• Activity patterns for the faces versus scenes attentional states became more separable after training as assessed by MVPA • Sustained attention abilities improved in participants who received NF training	High
Habes et al. (2016)	PPA/FFA; functional localizer	9 young healthy adults, (8 controls)	• 6 runs of 3 min each, 1 day • Continuous thermometer • No feedback for the controls, training in a mock scanner	• Successful upregulating differential PPA/FFA activity • Binocular rivalry task performance showed no behavioural changes after training	High
Robineau et al. (2014) and Robineau, Meskaldji, et al. (2017)	Visual areas in left and right occipital cortex; functional localizer	14 young healthy adults (no controls)	• 3 runs of 60 min each, 3 days • Continuous thermometer	• Consistent up-regulation of the target ROI activity in 8/14 participants • No significant improvement in bilateral target detection task and line bisection task (Robineau et al. 2014) • The successful learners achieved similar activity levels 14 months after the training without any neurofeedback (Robineau, Meskaldji, et al. 2017)	Moderate
Robineau, Saj, et al. (2017)	Unilateral right V1/bilateral V1; functional localizer	9 elderly chronic stroke patients with left hemispatial neglect (2 experimental groups with 6 and 3 participants)	• 12–15 runs of 3 min each, 3 days over 3 weeks • Auditory feedback between 0 (lowest) and 10 (highest) on ipsilesional V1 activity (unilateral group) or differential V1 feedback (bilateral group) every 6 s	• No effects in the bilateral group, positive results in the unilateral group • Significant increase in activity levels over the training sessions • Recruitment of bilateral frontoparietal areas, increased localization to the contralesional hemisphere over the sessions • Significant decrease in errors in the line bisection task between the pre-training and session 3, significant reduction of neglect severity according to conventional tests taken pre- and post-training	High
Rota et al. (2009)	Right IFG; anatomical and functional localizers	12 young healthy adults (5 controls)	• 4 runs of 9 min each, 1 day • Continuous thermometer • Sham NF from unrelated regions	• Progressive increase in ROI activation specific to the NF group • Improvements in the experimental group in interpreting emotional prosody but not syntax	High
Scharnowski et al. (2012)	Early visual cortex representing the left or right visual field; functional and anatomical localizer	16 young healthy adults (5 controls)	• 6 runs of 8.3 min each, 3 days; • Continuous thermometer • Sham NF from an unrelated region	• Significant increases in visual cortex activity in 7/11 experimental participants • Associated increase in connectivity between the visual cortex and the superior parietal lobe • Significantly enhanced perceptual sensitivity in successful learners	High
Sherwood et al. (2016)	Left DLPFC activity; functional localizer	25 young healthy adults (7 controls)	• 5 runs of 8 min each, 5 days over 2 weeks • Continuous line graph • No feedback information for the controls	• Ability of ROI activity regulation significantly increased in the experimental group • Associated increase in working memory performance assessed with the 2-back task and dual-task scenario	High
Shibata et al. (2011)	V1/V2; functional localizer for fMRI decoder	10 young healthy adults (no controls)	• 10 runs of 5 min each, 5–10 days • Intermittent, solid green disk	• Learned estimation of target-orientation likelihood, even during the first neurofeedback day • Performance in orientation discrimination task significantly improved	Moderate
Zhang et al. (2013)	Left DLPFC; functional localizer	30 young healthy adults (15 controls)	• 8 runs of 6.5 min each, 2 days • Continuous thermometer • Sham NF from the experimental group	• ROI activity significantly increased between the first and last training session • Experimental group showed improved performance on the digit span and letter memory task	High

a Abbreviations in alphabetical order: DLPFC, dorsolateral prefrontal cortex; FFA, fusiform face area; M1, primary motor cortex; MVPA, multi-variate pattern analysis; NF, neurofeedback; PHC, parahippocampal cortex; PMC, premotor cortex; PMv, ventral PMC; PPA, parahippocampal place area; ROI, region of interest; RT, response time; S1, primary somatosensory cortex; SMA, supplementary motor area; V1/V2, primary/secondary visual cortex.

b Where applicable, the number of controls is included in the total number.

c The age of young adults ranged from 18 to 46; the age of elderly adults ranged from 41 to 77.

### 3.1 Quality assessment

According to the JBI criteria, two studies were deemed of low quality and 14 studies of moderate quality. The remaining 17 were rated as high-quality studies. Noteworthy, only few studies assessed the long-term effects of the neurofeedback training on the participants (Table 3).

**Table 3:** Quality assessment of the included studies based on the JBI checklist for semi-experimental studies, which includes 9 criteria (established temporal relationship between the variables; similar participants; similar treatment; control group; multiple outcome measurements; follow-up; similar outcome. measurements; reliable outcome measurements; and appropriate statistical analysis).

Study	1. Cause and effect	2. Similar participants	3. Similar treatment	4. Control group	5. Multiple outcome measures	6. Follow-up	7. Similar outcome measures	8. Reliable outcomes	9. Appropriate statistical analysis	Score
**Studies with neural measures only**										
*Motor domain*										
Auer et al. (2015)	1	1	0	1	1	0	1	1	1	7
Berman et al. (2012)	1	0	0	0	1	0	0	1	1	4
DeCharms et al. (2004)	1	1	1	1	0	0	1	1	1	7
Hampson et al. (2011)	1	0	0	0	1	0	0	1	1	4
Johnson et al. (2012)	0	1	0	0	0	0	1	1	0	3
Liew et al. (2016)	1	0	0	0	1	0	1	1	1	5
Marins et al. (2015)	1	1	1	1	0	0	1	1	1	7
Neyedli et al. (2017)	1	0	0	1	1	0	1	1	1	6
Perronnet et al. (2017)	1	0	0	0	1	0	1	1	1	5
Xie et al. (2015)	1	1	1	1	1	0	1	1	1	8
Yoo and Jolesz (2002)	1	0	0	0	0	0	0	1	0	2
Yoo et al. (2008)	1	1	1	1	1	1	1	1	1	9
*Cognitive domain*										
Banca et al. (2015)	1	0	0	0	1	0	0	1	1	4
Ramot et al. (2016)	1	0	0	0	1	0	0	1	1	4
Yoo et al. (2006)	1	1	1	1	1	0	1	0	1	7
Yoo et al. (2007)	1	1	1	1	1	1	1	1	1	9
**Studies with behavioural and neural measures**										
*Motor domain*										
Blefari et al. (2015)	1	0	0	0	1	0	0	1	1	4
Bray et al. (2007)	1	1	1	1	1	0	1	1	1	8
Chiew et al. (2012)	1	1	1	1	1	0	1	1	1	8
Hui et al. (2014)	1	1	1	1	1	0	1	1	1	8
Scharnowski et al. (2015)	1	0	0	0	0	0	0	1	1	3
Sitaram et al. (2012)	1	0	1	0	1	0	1	1	1	6
Zhao et al. (2013)	1	1	1	1	1	0	1	1	1	8
*Cognitive domain*										
Amano et al. (2016)	1	1	1	1	1	1	1	1	1	9
DeBettencourt et al. (2015)	1	1	0	1	1	0	1	1	1	7
Habes et al. (2016)	1	1	0	1	1	0	1	1	1	7
Robineau et al. (2014) and Robineau, Meskaldji, et al. (2017)	1	0	0	0	1	1	0	1	1	5
Robineau, Saj, et al. (2017)	1	1	1	0	1	0	1	1	1	7
Rota et al. (2009)	1	1	1	1	1	0	1	1	1	8
Scharnowski et al. (2012)	1	1	1	1	1	0	1	1	1	8
Sherwood et al. (2016)	1	1	0	1	1	0	1	1	1	7
Shibata et al. (2011)	1	0	0	0	1	0	0	1	1	4
Zhang et al. (2013)	1	1	1	1	1	0	1	1	1	8

### 3.2 Modulation of brain activity and connectivity

Most of the studies in healthy individuals showed successful regulation of brain activity in the target ROI, or of the functional connectivity between two or more target ROIs; six studies reported no neural effects of rt-fMRI neurofeedback at the group level (Blefari et al., 2015, Chiew et al., 2012, Johnson et al., 2012, Ramot et al., 2016, Robineau et al., 2014, Scharnowski et al., 2012) (Table 1, Table 2).

Three studies in the cognitive domain (Amano et al., 2016, Robineau et al., 2017, Yoo et al., 2007) and one study in the motor domain (Yoo, Lee, O'Leary, Panych, & Jolesz, 2008) followed up on the participants after the rt-fMRI neurofeedback training over long periods (between 2 weeks and 14 months). The results suggest that the ability to self-modulate brain activity can be preserved up to 14 months after the initial neurofeedback training.

The studies that applied rt-fMRI neurofeedback to ameliorate stroke-induced behavioural impairments provided evidence that stroke patients can modulate the neural activity in, and connectivity between brain areas implicated in the impaired functions. In Liew et al. (2016), stroke patients learned to modulate functional connectivity between the primary motor cortex and the thalamus in the ipsilesional hemisphere. Half of the patients were able to maintain control of this cortical–subcortical connectivity during the transfer run, and all showed an increased resting-state connectivity between the two regions following the training. Sitaram et al. (2012) successfully used rt-fMRI neurofeedback on the ventral premotor cortex to remediate mild upper limb motor impairments in chronic stroke survivors. After three days of training, three times a day, the patients were able to regulate activity in the ventral premotor cortex, and maintained it during the transfer run. In Robineau, Saj, et al. (2017), patients with hemispatial neglect were able to control activity in the ipsilesional early visual cortex, but not the differential activity between the contra- and ipsilesional early visual cortex.

### 3.3 Training-induced behavioural modulation of motor function

In almost all studies using rt-fMRI neurofeedback to train motor function (Blefari et al., 2015, Bray et al., 2007, Chiew et al., 2012, Hui et al., 2014, Scharnowski et al., 2015, Sitaram et al., 2012, Zhao et al., 2013), experimenters encouraged the participants to perform motor imagery as a strategy to self-regulate cortical activity. Studies in healthy participants reported neurofeedback-related improvements in motor performance when participants were trained to regulate activity in the supplementary motor area, the sensorimotor cortex, and the ventral and dorsal premotor cortex. Similarly, Sitaram et al. (2012) showed improvements in visuomotor functioning following rt-fMRI neurofeedback training on the ventral premotor cortex in stroke survivors with right hemiparesis. No significant behavioural change was found in the studies aimed at regulating activity in the primary motor area (Blefari et al., 2015, Chiew et al., 2012) (Table 2).

### 3.4 Training-induced behavioural modulation of cognition

A substantial number of studies assessing the effect of rt-fMRI neurofeedback on cognitive performance were in the domain of visual perception. Most of these targeted early visual areas V1 and V2 (Amano et al., 2016, Robineau et al., 2014, Robineau et al., 2017, Scharnowski et al., 2012, Shibata et al., 2011), whereas one study targeted the higher visual areas parahippocampal place area and fusiform face area (Habes et al., 2016). Four of these studies observed behavioural changes after the training (Table 2). For instance, Robineau, Saj, et al. (2017) showed that rt-fMRI neurofeedback training can reduce symptoms of hemispatial neglect in chronic stroke patients. Consistent with the observations at the neural level (Section 3.2), the study reported a reduction of hemispatial neglect assessed with a line bisection task when participants learned to upregulate ipsilesional visual cortex activity. This is the first neurofeedback study to suggest that exerting control over the activity in the ipsilesional visual cortex may enable stroke patients to reduce the spatial attention bias characteristic of hemispatial neglect.

The rt-fMRI neurofeedback studies focussing on other cognitive functions were all performed in healthy participants. They reported an improved behavioural performance after rt-fMRI neurofeedback training (DeBettencourt et al., 2015, Rota et al., 2009, Sherwood et al., 2016, Zhang et al., 2013). Zhang et al. (2013) and Sherwood et al. (2016) reported an improvement in working memory performance after neurofeedback training to modulate dorsolateral prefrontal cortex activity. DeBettencourt et al. (2015) developed a sustained attention training paradigm using rt-fMRI neurofeedback, and behavioural performance in a go/no-go task improved after just one training session. Finally, Rota et al. (2009) examined how emotion processing by the right inferior frontal gyrus influenced language and speech processing, and showed that increasing activity in this region was correlated with improvements in interpreting emotional prosody in a linguistics task, but not in a syntax task.

## 4 Discussion

Previous research demonstrated correlational links between brain function and behaviour, and the use of neurofeedback enabled causal links to be substantiated through the voluntary modulation of one's own brain activity. With the correct strategy, this knowledge can be used by clinicians to ameliorate behavioural deficits by facilitating endogenous control over brain activity, likely with higher specificity and fewer side effects than pharmaceutical therapies (Weiskopf, 2012). Accumulating evidence suggests the efficacy of rt-fMRI neurofeedback in the treatment of neurodevelopment and neuropsychiatric disorders, such as attention deficit disorders, anxiety, depression, addictions, and autism spectrum disorders (for a review, see Stoeckel et al., 2014). However, rt-fMRI neurofeedback is still relatively new in the field of stroke rehabilitation. Due to the high costs of MR scanning, rt-fMRI is expected to become a clinically used approach only if it is proven that it can bring clear benefits to the patients' quality of life. To address this issue, here we conducted a systematic review assessing the potential of rt-fMRI neurofeedback for the rehabilitation of motor and cognitive impairments following stroke. Effective modulation of cognitive and motor performance through self-regulated brain activity was shown in some – but not all – rt-fMRI neurofeedback studies conducted so far in healthy individuals and stroke patients. It should be noted that only three neurofeedback studies on stroke patients met the inclusion criteria (Liew et al., 2016, Robineau et al., 2017, Sitaram et al., 2011). Accordingly, the effectiveness of this non-invasive therapy for stroke rehabilitation needs to be more extensively evaluated.

### 4.1 Effects of rt-fMRI neurofeedback on brain function and behaviour

Overall, the findings from studies conducted in healthy individuals suggest that neurofeedback training has the potential to improve performance in motor and cognitive functions. At the same time, we observed that the effectiveness of real-time fMRI neurofeedback varies considerably across target regions. For instance, most of the studies targeting early visual areas showed significant neural or behavioural effects after the training (Banca et al., 2015, Robineau et al., 2014, Scharnowski et al., 2012, Shibata et al., 2011). In contrast, the studies targeting higher visual areas did not observe any significant effects (Habes et al., 2016, Ramot et al., 2016). Also, successful regulation has been observed in most of the studies targeting the sensorimotor or premotor cortex (Auer et al., 2015, Bray et al., 2007, DeCharms et al., 2004, Hui et al., 2014, Zhao et al., 2013), but limited success has been obtained in modulating primary motor cortex activity through motor imagery (Berman et al., 2012, Blefari et al., 2015, Chiew et al., 2012; however, see; Perronnet et al., 2017, Yoo et al., 2008). Noteworthy, it's still a matter of debate whether primary motor cortex is recruited during motor imagery (Sharma, Pomeroy, & Baron, 2006).

For stroke patients, rehabilitation protocols that do not require the patients to make overt movements, which is the case for neurofeedback-based training, may be beneficial since prolonged physical effort can be avoided. The results of this systematic review indicate that stroke patients, like healthy individuals, can learn to control brain activity through neurofeedback, and this might ultimately lead to an improvement of stroke symptoms. This postulation is also confirmed by studies aiming at modulating brain activity and connectivity in stroke with fNIRS (Mihara et al., 2012, Mihara et al., 2013), MEG (Boe et al., 2014, Buch et al., 2012), or EEG (Ramos-Murguialday et al., 2014, Shindo et al., 2011, Young et al., 2014). Notably, evidence exists for a successful use of EEG neurofeedback for cognitive and motor rehabilitation in stroke, but the effects are not consistent across participants (Bearden et al., 2003, Cannon et al., 2010, Doppelmayr et al., 2007, Reichert et al., 2016, Rozelle and Budzynski, 1995). We posit that, thanks to its superior spatial resolution, rt-fMRI can provide more accurate feedback than EEG/MEG and fNIRS to the participants, who may more easily learn to control their brain activity or connectivity.

### 4.2 Important factors for the design of rt-fMRI neurofeedback studies

*Use of control groups/treatments*. The use of appropriate control treatments is particularly important when assessing behavioural changes induced by rt-fMRI neurofeedback training. In this regard, it should be noted that about one third of the reviewed studies did not include a control group, and this impedes quantitative analyses concerning the effectiveness of the intervention. Studies with a control group mostly included sham-feedback groups, where feedback was presented based on brain activity recorded in a different participant (e.g., Hui et al., 2014, Xie et al., 2015, Zhang et al., 2013) or from a brain region of the same participant but unrelated to the function of interest (e.g., DeCharms et al., 2004, Neyedli et al., 2017, Rota et al., 2009, Yoo et al., 2007, Yoo et al., 2008). Others included no-neurofeedback behavioural training groups either inside or outside the MR scanner (e.g., Amano et al., 2016, Auer et al., 2015, Yoo et al., 2006). Taken together, the use of sham-neurofeedback was crucial to demonstrate the importance of real, contingent neurofeedback in learning to modulate brain activity in a wide range of brain regions (DeCharms et al., 2004, Linden and Turner, 2016, Scharnowski and Weiskopf,, Sepulveda et al., 2016). However, the effectiveness of rt-fMRI neurofeedback-based therapy for stroke rehabilitation is still to be compared to conventional stroke therapy. Likewise, comparisons between experimental groups with different demographics might reveal factors that influence both the ability to learn self-regulation and the emergence of behavioural effects (e.g., age, Neyedli et al., 2017). To this end, carefully designed rt-fMRI neurofeedback studies should be conducted in stroke patients and age-matched control subjects, as already done in EEG neurofeedback studies (Becerra et al., 2011, Cho et al., 2016, Kober et al., 2015, Shindo et al., 2011, Staufenbiel et al., 2014).

*Potential biases in participant allocation*. Most of the studies were single-blinded and validated in the sense that participants who did not receive real feedback did not notice it, or were unaware that the experiment involved multiple groups of participants. The degree of blinding of the experimenter during the training and of the assessor during the post-training assessment was not specified, with an exception of two studies. Notably, these studies implemented a double-blind procedure by letting a different researcher conduct the participant recruitment and scheduling (DeBettencourt et al., 2015, Neyedli et al., 2017). This double-blind procedure would be an appropriate approach for the unbiased assessment of rt-fMRI neurofeedback effects (Stoeckel et al., 2014), in particular in randomized control trials.

*Duration/intensiveness of the training*. Almost all studies that failed to find an effect of rt-fMRI neurofeedback on behavioural performance, also did not show clear signs of neural modulation (Blefari et al., 2015, Chiew et al., 2012, Robineau et al., 2014). Studies without behavioural effects were typically conducted within a single day, though there does not seem to be a strong link between the absence of effects at the neural level and training duration (ranging from 1 to 7 days). Other studies with short training protocols showed significant pre-post behavioural learning effects after sessions as short as 30 min (Hui et al., 2014, Zhao et al., 2013), and neural effects after a single run of 8 min (Yoo & Jolesz, 2002). These results are promising for clinical applications of rt-fMRI neurofeedback, possibly in combination with other interventions outside the scanner (Yoo et al., 2007, Yoo et al., 2008). Most studies with multi-day sessions showed increasing control of ROI activity over the course of the training. None of the reviewed studies compared the magnitude of the learning effects across training days to that from multiple training sessions on the same day. However, other neurofeedback studies have found a sleep consolidation effect where performance increased significantly more between training days compared to between runs on the same day (Megumi et al., 2015, Scheinost et al., 2013). Although no golden standard exists, it has been suggested that successful transfer of learned self-regulation in the absence of neurofeedback can be expected if at least half of the training runs reach a significantly increased activation (Auer et al., 2015).

*Training design*. Almost all studies made use of a block design, alternating blocks aimed at regulating neural activity with resting-state blocks. In contrast, Banca et al. (2015) built in a semi-event-related feature that allowed the participant to ‘self-pace’ the training by choosing the order and the duration of the blocks. This self-paced design could potentially improve participant engagement and increase the effectiveness of the training. Notably, the optimal way to learn to control one's own brain activity varies greatly between participants. For example, Scharnowski et al. (2015) found that explicit cognitive strategies worked best in facilitating neurofeedback learning over the supplementary motor area, whereas Sepulveda et al. (2016) showed best learning effects in the same region when providing a monetary reward without explicit instructions. Further investigations on the mechanisms of operant conditioning in neurofeedback paradigms are warranted for the design of protocols that can give rise to successful learned self-regulation of the targeted brain activity (Birbaumer et al., 2013, Sulzer et al., 2013).

*Type of feedback*. In general, the type of neurofeedback given can vary in modality (auditory and visual feedback), degree of processing (presenting raw brain activity or a derived measure), and timing of the presentation (continuous or intermittent). A few studies gave feedback in the auditory modality (Banca et al., 2015, Ramot et al., 2016, Robineau et al., 2017, Yoo et al., 2006), whereas the majority provided visual feedback. The feedback is typically designed to minimize distraction from the task at hand, however, the effect of feedback modality on training efficacy has not been systematically investigated (Emmert et al., 2016). About half of the studies that trained visual perception opted for auditory feedback since it is in a different modality. Regardless of the modality, the majority of the studies provided continuous feedback such that subjective experience could be linked to a ‘tangible’ output. Interestingly, Johnson et al. (2012) showed that participants preferred intermittent compared to continuous feedback in a motor imagery task. The authors suggested that intermittent feedback is more effective in promoting self-regulation of activity in the premotor cortex, however, this study did not directly compare the effect of continuous versus intermittent feedback on training efficacy. There are no best-practice guidelines in the literature concerning the use of continuous or intermittent feedback (Sulzer et al., 2013).

*Behavioural outcome measures*. The reviewed studies that investigated behaviour included a variety of outcome measures chosen to fit the experimental paradigms of each study. An important factor to consider is the sensitivity of the outcome measures used: if a study does not report significant behavioural changes, it may simply be due to the fact that the outcome measure is not sufficiently sensitive. From this perspective, recent studies have highlighted the importance of using computerized tests for a refined quantification of the participants' performance on a variety of motor and cognitive tasks (Bonato and Deouell, 2013, Nordin et al., 2014, Pedroli et al., 2015). Also, the use of standardized neuropsychological test batteries suitable for stroke patients may be particularly helpful. They enable qualitative and quantitative comparisons across experiments using different brain areas as targets for rt-fMRI neurofeedback, and allow for predictions on quality of life following the training (Bickerton et al., 2014, Demeyere et al., 2015, Fugl-Meyer et al., 1975). Overall, the use of reliable indices that can show clinically significant changes due to the training is crucial to the development of rt-fMRI neurofeedback as a novel therapeutic tool (Stoeckel et al., 2014).

*Follow up/transfer*. The current review included only a limited number of long-term follow-up studies in healthy participants, and none in stroke patients (Amano et al., 2016, Robineau et al., 2017, Yoo et al., 2007, Yoo et al., 2008). Previous fNIRS and EEG studies investigating the long-term effects of neurofeedback in stroke patients show that the improved motor function could be retained up to four weeks after training (Mihara et al., 2013, Mottaz et al., 2015), but this has not yet been shown for rt-fMRI neurofeedback training in stroke patients.

### 4.3 Caveats and future directions

The findings of this systematic review suggest that rt-fMRI neurofeedback may be effective in ameliorating motor and cognitive deficits in stroke patients. Nonetheless, the limited number of patient studies does not allow to draw conclusions about the efficacy and effectiveness of this technique, which still need to be thoroughly evaluated in future studies. The use of different neurofeedback training approaches and outcome measurements complicates any direct comparison between studies aimed at ameliorating the same function. Although no golden standard exists for the assessment of cognitive and motor impairments in stroke patients, a more consistent selection of methods would ease the comparison of the results obtained across studies. Based on the considerations above, we suggest that future double-blind randomized experiments should include a relatively large number of stroke patients to permit group-level inferences about the efficacy of rt-fMRI neurofeedback. Also, systematic outcome measures on behavioural functions should be used, possibly relying on a standardized battery of clinically-relevant tests (Bickerton et al., 2014, Demeyere et al., 2015, Fugl-Meyer et al., 1975). Finally, both short- and long-term effects of rf-fMRI neurofeedback should be assessed in follow-up studies to shed light on the degree with which neurofeedback can trigger sustained changes in brain activity and consequently, behaviour.

## 5 Conclusion

Effective rehabilitation approaches to improve motor and cognitive function of stroke patients are still lacking. The results emerging from this systematic review suggest that rt-fMRI neurofeedback permits self-regulation of brain activity and can lead to behavioural effects. As such, a more widespread application in the field of stroke rehabilitation is warranted. Neurofeedback may prove particularly useful in early stages after stroke, when physically strenuous interventions are not possible or recommended. In particular, neurofeedback can show the participants that they can take control over seemingly volitionless aspects of their impairment. This feeling of increased control will most likely benefit the individual through the recovery process. Additionally, neurofeedback training in the chronic stage of stroke, where spontaneous recovery has stopped, may trigger functional reorganization in structurally intact parts of the brain, possibly leading to a behavioural recovery that would otherwise not occur.
